# Addressing Magnesium Deficiency Through Crop Biofortification: Plant–Soil–Human Perspective—A Review

**DOI:** 10.3390/plants15050801

**Published:** 2026-03-05

**Authors:** Jan Vašíček, Martin Kulhánek, Kateřina Šulcová, Jan Hladík, Jindřich Černý, Jiří Balík

**Affiliations:** Department of Agro-Environmental Chemistry and Plant Nutrition, Faculty of Agrobiology, Food and Natural Resources, Czech University of Life Sciences, 165 00 Prague, Czech Republic; vasicekj@af.czu.cz (J.V.); sulcovakaterina@af.czu.cz (K.Š.); hladikjan@af.czu.cz (J.H.); cernyj@af.czu.cz (J.Č.); balik@af.czu.cz (J.B.)

**Keywords:** magnesium deficiency, fortification, soil, fertilizers, plant breeding

## Abstract

Magnesium is an essential macronutrient for both plants and humans. However, its availability in agricultural systems and dietary intake has been declining, raising concerns about crop productivity and nutritional security. In plants, magnesium plays a critical role in photosynthesis, enzyme activation, carbohydrate transport, and overall metabolic regulation, while in humans it is required for numerous biochemical processes related to energy metabolism, cardiovascular function, and disease prevention. Long-term studies have reported a 20–30% decrease in magnesium concentrations in fruits and vegetables worldwide, potentially contributing to widespread magnesium deficiency. Soil factors such as acidification, nutrient imbalance, and intensive agricultural practices further limit magnesium availability along the soil–plant–human continuum. This review summarizes the biological importance of magnesium in plants and humans, evaluates the occurrence and causes of magnesium deficiency, and discusses current strategies for improving magnesium nutrition through agronomic and genetic biofortification. It considers even fertilizer management, nano-fertilizers, and alternative magnesium sources such as serpentinite. The review highlights biofortification as a cost-effective and sustainable strategy to enhance crop magnesium concentration and mitigate global magnesium deficiency while emphasizing the need for further research on bioavailability, environmental safety, and long-term agricultural sustainability.

## 1. Introduction

This review is based on the hypothesis that magnesium deficiency represents an underrecognized bottleneck linking soil degradation, impaired crop nutritional quality, and human health outcomes, and that targeted agronomic biofortification strategies can effectively mitigate this soil–plant–human continuum. Magnesium (Mg) is an essential macro-element, critically important for plant growth and development. It plays a vital role in various physiological and biochemical processes, including photosynthesis, chlorophyll synthesis, enzyme activation, protein synthesis, and stabilization of nucleic acids [1]. Experimental studies have consistently shown that magnesium deficiency disrupts photosynthetic carbon assimilation, carbohydrate partitioning, and phloem transport, ultimately restricting biomass formation and agricultural productivity [2]. A systematic evaluation of more than 70 years of experimental data demonstrated that adequate magnesium supply can increase net CO_2_ assimilation by up to 140% and plant biomass production by more than 60% compared with Mg-deficient conditions [3].

Magnesium also influences nutritional quality as well as yield. However, over the past six decades, various studies have shown a 20% to 30% reduction in the concentration of this macronutrient in fruits and vegetables [1,4]. Magnesium deficiency can disrupt plant metabolism and reduce overall output [5]. Agronomic biofortification, a method to enhance the content of mineral elements in edible crop organs through agronomic interventions, is an appealing option to mitigate magnesium deficiency in human diets [6]. Biofortification of cereal and grain staple crops is on the rise, with breeding programs increasingly directed towards enhancing nutritional quality [7].

Magnesium nutrition has received increasing attention in recent years due to its measurable impact on agricultural productivity. A global meta-analysis based on 570 field observations (99 field research articles) demonstrated that magnesium fertilization increased crop yield by approximately 8.5% across diverse crops and soil conditions, highlighting Mg as a frequently limiting nutrient in modern production systems [5].

As it is vital to plants, it is also necessary for human health. Without adequate levels of magnesium, individuals may initially experience loss of appetite, nausea, weakness, and mood swings [8]. Nonetheless, Mg deficiency has been associated with several diseases, such as diabetes, asthma, depression, Alzheimer’s disease, bone fragility, and more [9]. It is estimated that 45% of Americans suffer from Mg deficiency [10], while in Europe it affects around 10–15% of the population [11].

Biofortification is a cost-effective and sustainable way of reducing nutrient malnutrition, especially among populations highly dependent on staple foods [12]. Agronomic biofortification, specifically, employs the use of mineral fertilizers to increase the levels of target elements in crops [13]. The advantage of biofortification lies in its potential to reach remote rural areas with little access to commercially fortified foods, complementing other interventions like commercial fortification and supplementation programs [14].

Biofortified crops can be integrated into the existing agricultural system, supplying nutrients regularly to consumers. Initial investment in agricultural research yields high recurrent returns as new varieties become available in different areas and prove to be low-cost and effective. Although essential roles of magnesium in numerous vital processes are well recognized, research into how magnesium nutrition impacts crop yield and quality remains relatively rare. The objective of this review is to provide information about the biological importance of magnesium, the types of biofortification, and its effects on human health.

## 2. Biological Importance of Magnesium

### 2.1. In Plants

Magnesium is an indispensable component in many crucial physiological and biochemical processes throughout plant growth and development. It takes part in more than 300 enzymatic reactions. One of the most important enzymes is Rubisco, functioning in the Calvin cycle. Other important enzymes include protein kinases, RNA polymerase, glutathione synthase, ATPases, phosphatases, and carboxylases. In general, Mg^2+^ is essential for nearly all ATP-dependent enzymes in plants, as it forms the actual substrate (Mg-ATP) recognized by pumps, kinases, and synthases. However, the real key to autotrophic development is Rubisco’s Mg-mediated activation [15,16].

Magnesium accounts for approximately 2.7% of the mass of a chlorophyll molecule and is necessary for the formation and maintenance of proper chloroplast structure. According to Marschner [17] up to 35% of the Mg^2+^ in plants is bound in chlorophyll. Magnesium is very mobile in the phloem, and loading is typically sustained so long as internal leaf Mg content is sufficient, independent of external supply. Nonetheless, phosphate supply has been shown to increase mobilization of Mg and phloem loading. Importantly, Mg participation in phloem loading is highly specific and quickly reversible because sucrose export from leaves can be quickly restored following resupply of magnesium to stressed plants [18].

Uptake of magnesium occurs by roots via specific transport systems including MRS2/MGT (magnesium transporter) which are homologous to bacterial *CorA* Mg^2+^ transporters and represent the major Mg transporter family in higher plants such as rice, maize, *Arabidopsis thaliana* and other crop species [19]. These MRS2/MGT transporters are found in plasma and organellar membranes such as the chloroplast and the mitochondria where they help with Mg^2+^ uptake and maintain magnesium balance inside the cells [20]. Additionally, studies indicate that several MRS2/MGT genes are activated in response to magnesium deficiency and other stress conditions. This shows how magnesium acquisition and distribution are regulated when magnesium levels change [21].

For healthy growth, Rengel et al. [15] report that plants need about 1.5–3.5 g Mg per kg of biomass, whereas values below ~1.0–1.5 mg g^−1^ (dry weight) are frequently associated with reductions in photosynthesis. Content of Mg in plants, however, heavily differs depending on many factors, such as species, part of plant, fertilization, soil type, etc. [22]. Table 1 presents the average Mg concentrations in different agricultural crops. The data clearly indicate that leafy vegetables exhibit the greatest potential for Mg biofortification. From the perspective of cereal crops, the consumed grain fraction must be considered. The highest proportion of magnesium (up to 40%) is accumulated in the aleurone layer [23]. Elevated concentrations are also found in the germ [24] and bran fractions, whereas the content in the starchy endosperm is comparatively low [25]. However, it is necessary to consider even Mg bioavailability for humans as discussed further in Section 5.2.

Chlorosis is one of the first and most common symptoms of magnesium deficiency, typically appearing initially in older leaves before progressing to younger tissues. However, a lack of magnesium for chlorophyll synthesis is not the only reason why plants with magnesium deficiencies experience a decrease in chlorophyll content. Instead, it seems that magnesium deficiency affects the leaves’ overall protein synthesis, which in turn limits the production of chlorophyll [45]. Yellowing results from disrupted chlorophyll production and increased oxidative stress, often leading to leaf wilting and the appearance of necrotic spots [46,47,48].

Studies have demonstrated that Mg-deficient plants accumulate substantial amounts of non-structural carbohydrates in source leaves, accompanied by reduced translocation to roots and developing reproductive organs, leading to sink limitation and feedback inhibition of photosynthesis [49,50]. This feedback inhibition further exacerbates the decline in carbon assimilation. Notably, the inhibition of sucrose export is rapidly reversible upon Mg resupply, underscoring a highly specific and dynamic regulatory role of Mg in phloem loading and long-distance transport [49,50,51].

### 2.2. In Human Body

Magnesium is a necessary and frequently overlooked mineral responsible for many biochemical reactions fundamental to human health, impacting processes from energy production to genomic stability. It is the fourth most common mineral in the human body, with a content ranging from 25 to 50 g, and is an essential cofactor in more than 300 enzymatic reactions [52,53].

Magnesium is also essential for many physiological functions, such as protein synthesis, cellular respiration, and maintenance of membrane integrity [53]. This mineral plays a crucial role in the storage, replication, and repair of genetic information because it maintains the structural integrity of nucleic acids, including DNA and RNA [8,54]. Magnesium has a wide-ranging impact on metabolic function, as evidenced by its involvement in muscle contraction, nerve impulse conduction, and blood glucose regulation [55]. Magnesium consumption is crucial for preserving overall health and preventing chronic diseases [11]. The daily magnesium requirement is approximately 420 mg for males and 320 mg for females [52]. The human body contains around 25 g (approximately 1000 mmol) of magnesium, with about 98% stored in soft tissues (38%) and bones (60%) [56,57].

Staple crops are the primary sources of magnesium for humans; therefore, it is necessary to maintain optimal conditions for these crops, including soil factors that have a significant impact on magnesium concentrations [58]. A significant proportion of the population in the Western world fails to achieve the recommended daily allowance for magnesium, potentially contributing to various adverse health conditions [52]. Mg plays a key role in regulating vascular function, glucose homeostasis, and oxidative stress. Low intake of magnesium is associated with various chronic diseases including hypertension, cardiovascular disease, metabolic syndrome, and insulin resistance [59,60,61]. This finding is consistent with the population study by [62], which reported that individuals with higher dietary magnesium intake had approximately 15% lower risk of developing type 2 diabetes.

Magnesium also contributes to endocrine regulation, including the reproductive axis. Some studies indicate that adequate magnesium levels might contribute to higher testosterone levels, possibly due reduced oxidative stress and modulation of sex hormone-binding globulin (SHBG) binding. Although evidence is still limited, magnesium deficiency could be an overlooked contributor to hormonal imbalance [63,64]. In the human diet, legumes and beans (~1450–1710 mg kg^−1^ DW) are two of the most dependable sources of magnesium. Magnesium is also abundant in nuts and seeds, especially pumpkin (~5500–5900 mg kg^−1^ DW) and chia seeds (3350 mg kg^−1^ DW), as well as almonds (2860 mg kg^−1^ DW). Reliable sources of this vital mineral include whole grains such as oats, whole-grain bread, and other unprocessed cereals (~1210–1770 mg kg^−1^ DW). Compared to other foods, oranges are not particularly magnesium-dense, but they do contain a small amount among fruits. Even animal foods such as milk (~850 mg kg^−1^ DW), dairy products, fish, and meat also contribute magnesium to the diet, with milk being particularly significant in some dietary patterns [65,66].

Magnesium content in staple foods like wheat, maize, rice, and vegetables has tended to decline over the decades, with more recent varieties containing less magnesium than older ones. In the last 80 years (1940–2019), content of magnesium in fruit and vegetables declined by 10% [67]. An overall decline in nutrients has been confirmed by many other studies from various countries, which confirm a decline in magnesium up to 16–24% regarding not only staple crops, but fruits and vegetables as well [68,69,70]. Sorghum, maize, and wheat are important dietary sources of magnesium in some regions. Magnesium content is mainly influenced by soil, agricultural practices, and crop type [16,71]. Magnesium-related nutritional disorders in humans are most likely associated with decreasing magnesium levels in soils and agricultural crops [72].

## 3. Magnesium in Soil

Magnesium deficiency is a widespread issue that limits crop production and quality, especially in regions with acidic soils, intensive cultivation, and unbalanced fertilization. Acidic, sandy, and highly weathered soils are the most susceptible, and Mg deficiency can significantly reduce farm productivity [73].

Mg is weakly bound in soils, making it highly susceptible to leaching, especially in sandy soils, with high water movement and in regions with heavy rainfall or irrigation [74]. Low soil pH also increases Mg leaching and restricts its availability to plants. A one-unit reduction in soil pH from neutral can halve soil Mg content. There is rising concern about Mg deficiency in Central Europe due to acidic conditions (pH < 6.5) [5,75]. Soil magnesium deficiency results in magnesium-deficient feed, raising the risk of hypomagnesaemia in ruminants—a widespread and economically important issue in European livestock farming [76]. Table 2 shows differences among bioavailable Mg contents in soils around the world. Content of Mg in Europe differs vastly, mostly depending on soil type and bedrock. The main problem, as referred previously, is insufficient fertilization and acidity of soils supporting Mg leaching [77]. On the other hand, soils in the USA generally seem to have enough Mg reserves, except for a few southern states with slight deficits—namely Florida, Georgia and South Carolina [78]. Africa is mostly poor on Mg with exchangeable Mg rates reaching from 5 to 20 mg kg^−1^ [79,80]. In South America, most of the Mg is bound in minerals, and tropical and subtropical locations suffer by leaching and water erosion. Although total Mg content in soil may be high, exchangeable fractions of magnesium are at low levels [81]. China faces similar problems as Europe, including soil depletion, acidification, and uneven magnesium distribution. The concentration of exchangeable Mg in Northern China ranges from approx. 200 to 400 mg kg^−1^, whereas in Southern China, it often drops to 40–90 mg kg^−1^. Consequently, approximately 55% of China’s arable land is considered Mg-deficient [74]. It can be concluded that the most significant threat is the decline in the proportion of soils with an adequate supply of plant-available magnesium. For example, in the Czech Republic, the content of plant-available magnesium has been monitored for more than 50 years. During this period, the proportion of soils with low magnesium content increased by more than one third (from 9% in 1971–1975 to 14% in 2017–2022) [82].

In some cases, soil Mg resources are simply depleted due to intensive agriculture. The use of high-yielding crops, together with excessive use of NPK fertilizers, accelerates Mg deficiency [85]. Accelerated Mg depletion when using NPK can result from P-Mg synergism, which promotes higher yields as long as a proper K:Mg balance is maintained [36,86]. Potassium has been found to negatively affect magnesium uptake in plant roots. This antagonistic interaction has been demonstrated in studies by [26,35,40], where increasing potassium fertilization rates led to decreased magnesium uptake by plants. To limit the inhibitory effect of potassium on magnesium uptake, there must be a balanced availability of the mentioned elements and an optimal K:Mg ratio. However, it is not simple to determine the optimum ratio, since it relies on a wide range of physical and chemical soil properties as well as some plant-related parameters. Overall, the preferred ratio is believed to be 1K:2Mg [15,87]. Imbalance in the K:Mg ratio is often caused by neglected fertilization with magnesium [88].

Mild magnesium deficiency may cause yield reductions of 8–11%, while more severe deficiencies may lead to even greater losses. Magnesium is essential for proper photosynthesis; when it is lacking, this process is disrupted, resulting in poor plant growth, reduced crop tolerance, and lower quality of the final product. Soils poor in Mg can be restored with the right fertilization plan, considering the soil type; on acidic soils this includes pH adjustment with dolomitic limestone. Yields and crop quality can be significantly improved [5,89].

## 4. Clinical Manifestations of Magnesium Deficiency

The main cause of magnesium deficiency is insufficient dietary intake. However, gastrointestinal losses also play a significant role, especially in conditions such as short bowel syndrome, malabsorptive syndromes (like Crohn’s disease and celiac disease), and chronic diarrhea. Magnesium deficiency can also result from prolonged nasogastric suctioning and protein-calorie malnutrition [90]. Some medications are also known to cause magnesium deficiency. Notably, proton pump inhibitors (PPIs), calcineurin inhibitors, diuretics, and epidermal growth factor receptor (EGFR) inhibitors are the primary drug classes linked to magnesium deficiency [1].

Early symptoms of magnesium deficiency are rarely observed, since the kidneys conserve magnesium efficiently by lowering its excretion. Testing a blood sample is the most commonly used marker, but it may not reliably reflect total body or tissue magnesium status [54,91]. Symptoms usually appear once the deficiency has already advanced. Muscle cramps, tremors, and nervousness are common in magnesium deficiency, while more serious complications include cardiac arrhythmias, especially in older or acutely ill hospitalized individuals [92].

Magnesium deficiency has been associated with many health problems in humans, including cardiovascular disease, hypertension and stroke, cardiometabolic syndrome and type 2 diabetes, airway constriction syndromes and asthma, depression, stress-related conditions and psychiatric disorders, Alzheimer’s disease, dementias, muscle diseases (such as muscle pain, chronic fatigue, and fibromyalgia), bone fragility, and cancer. Health problems may vary with age. In older people, symptoms may include asthenia (weakness), sleep disturbances, hyperemotionality, and cognitive impairment, which may be mistaken for age-related changes [9].

Low magnesium levels are often accompanied by biochemical markers in the body, such as reduced potassium and calcium levels, and certain blood lipid imbalances—reduced HDL (“good”) cholesterol and elevated total cholesterol. Newer and more advanced testing methods, such as measuring ionized magnesium, can identify deficiency earlier than the common total serum magnesium test [93].

## 5. Magnesium Biofortification: Agronomic and Nutritional Aspects

Biofortification is the enhancement of the nutritional value of crops by using agronomic methods, traditional breeding techniques, or modern biotechnological approaches to increase the concentration and bioavailability of essential nutrients, vitamins, and minerals [94]. Increasing magnesium (Mg) in plants through biofortification is a good strategy to reverse endemic dietary Mg deficiency. There are two major approaches to biofortification: agronomic (fertilizer-based) and genetic (breeding or genetic modification). Both include an increase in Mg concentration and bioavailability in edible plant parts. Agronomic biofortification is a process involving the application of Mg fertilizer to the soil, or on top of plant leaves or stems, optimizing nutrient uptake, transport, and its concentration in the plant [95]. Generally, conventional fertilizers such as magnesium nitrate hexahydrate (Mg(NO_3_)_2_⋅6H_2_O), magnesium sulfate heptahydrate (MgSO_4_·7H_2_O), and magnesium chloride hexahydrate (MgCl_2_ ⋅6H_2_O) are applied to the soil or used as foliar sprays to increase Mg content in crops or vegetables such as beans and tomatoes. MgSO_4_·7H_2_O is noted to be very effective in increasing Mg and bioactive compound content in edible parts [13,96].

Foliar application is, however, most favored due to its ease of implementation, high use efficiency, and minimal requirements [97]. This type of application allows quick delivery of Mg directly to the leaves, where it can be absorbed and utilized by the plant while skipping xylem transportation of nutrients from the roots. Foliar applications are mostly used when there is a visible deficiency of magnesium [98]. Successful application of foliar fertilizer depends on several factors, such as the type of magnesium compound, spray concentration and timing, as well as weather conditions [5]. According to studies such as [99], foliar application can be as effective—or even more effective—than soil application. However, relying solely on foliar feeding is not considered a solution.

Amount of magnesium released from soil minerals can vary depending on soil texture, pH, and CEC; it is important to apply magnesium fertilizers to the soil to improve high crop yield and enhance the quality of the product [5,100].

Mineral magnesium fertilizers as well as compound fertilizers represent the primary source for replenishing Mg in soils (Table 3). These fertilizers differ mainly in solubility, release rate, amount of Mg content, and their effect on pH. Carbonate forms such as dolomite are used primarily on acidic soils where they additionally increase pH. Maintaining soil pH at an optimal level through systematic liming, for example by using dolomitic lime, can often effectively alleviate Mg deficiency in both soils and plants. Kieserite is highly soluble, and therefore it is a fast source for Mg-deficient crops. Organic fertilizers usually contain lower concentrations of Mg. They typically do not represent a rapid source of Mg, but they contribute to its long-term cycling in the soil and the general improvement of soil properties [101].

### 5.1. Nano-Fertilizers

Recent technological progress has resulted in the development of nano-fertilizers. This offers a new approach to improve nutrient delivery in crop production. Compared to conventional mineral fertilizers, nutrients in nano-form are taken up and utilized by plants more efficiently. This new method aims to boost fertilization precision while minimizing the environmental impacts typically associated with standard mineral fertilizers, which are often applied inefficiently, in improper doses, and often contribute to pollution [102,103,104]. Nano-fertilizers use nanoparticles to encapsulate essential nutrients like magnesium to improve nutrient uptake, control release, and increase plant growth and yield with lower application rates [105]. Results of various experiments suggest that these formulations not only support photosynthetic processes and modulate genes involved in nutrient uptake, but also strengthen plant resilience to stress [103]. This results in higher nutrient use efficiency, as well as crop productivity, while decreasing leaching, runoff, and potential phytotoxicity. Of course, this kind of fertilizer needs further investigation to fully understand how they interact with plants and soil, as well as to assess potential risks to human health and confirm their long-term environmental safety. Although there are still plenty of uncertainties, nano-fertilizers represent a promising strategy for advancing sustainable agriculture [104].

Although the majority of research has focused on nutrients like potassium, phosphorus, and nitrogen, Mg-based nano-fertilizers are gaining attention because of their potential to address crops that are low in magnesium [106]. Targeted nutrient delivery, decreased potential loss of nutrients, and improved plant growth are some of the benefits of nano-fertilizers [100]. The soil can be treated with nano-fertilizers; nonetheless, they are mostly used through foliar application [107]. The use of Mg nano-fertilizers is showing promising efficiency in increasing Mg content and improving nutritional quality compared to conventional fertilizers. For example, foliar application of Mg nano-fertilizer at 200 mg kg^−1^ resulted in over a 120% increase in Mg content in green beans, outperforming MgSO_4_ [95]. Mg-focused nano-fertilizers represent a sustainable step forward in precision agriculture, but further research is needed to fully realize their benefits and address regulatory and safety concerns [108].

Unfortunately, there is a very limited amount of data available on Mg nano-fertilizers as feed contaminants or environmental residue. A study by Abdelfattah [109] shows elevated liver enzymes (GOT, GPT) and altered renal markers indicating sub-clinical hepatic and renal stress in rats being fed seeds with NanoMgO treatment. MgO (bulk) may be chemically inert; however, the small size of nanoparticles allows deep penetration into biological systems, increasing its reactivity and biological interaction. Nongbet et al. [105] warns that nanotoxicity to soil biota, animals and humans is still in question. After Demeke et al. [110] a lot of countries still lack proper characterization and regulation of nano-fertilizers; therefore, their usage might seem hazardous.

Attention should be given to the potential accumulation and long-term effects of nano-fertilizers, as their altered physicochemical properties at the nanoscale may influence their bioavailability, plant metabolism, soil microbial communities, and potential transfer within the food chain. Therefore, comprehensive risk assessment and long-term monitoring are necessary to ensure the safe and sustainable implementation of nano-fertilizer technologies.

### 5.2. Bioavailability

It is necessary to mention that not all increased Mg in plants is equally bioavailable to humans. The form of Mg and the presence of other compounds affect its absorption [111]. Magnesium absorption in humans is regulated by both passive paracellular and active transcellular transport mechanisms and follows saturable kinetics [112]. For human intake, dividing magnesium into several smaller doses throughout the day seems to be better for its availability than a single large dose.

Certain dietary components are known to support magnesium absorption. They include proteins, medium-chain triglycerides, and various low- or indigestible carbohydrates such as resistant starch, oligosaccharides, inulin, mannitol, and lactulose [113,114].

Organic magnesium salts like magnesium citrate or lactate, naturally present in leafy greens (kale, lettuce, or arugula), could offer better absorption due to their higher solubility. An inorganic form such as magnesium chloride has good water solubility; therefore, it is comparable to magnesium citrate in terms of bioavailability [115].

Generally, studies show that the differences in bioavailability between organic and inorganic magnesium salts are minor and inconsistent [114,116].

In cereal grains, magnesium is primarily located in the aleurone and bran layer, where it is bound in the form of magnesium–phytate complexes. These complexes play a crucial role in limiting the bioavailability of magnesium for human nutrition because phytate is known to strongly inhibit the absorption of magnesium in the digestive tract [23,117]. The presence of phytate reduces the amount of magnesium that can be utilized by the body from consuming whole grains [118].

When the bran is removed during grain processing, the total magnesium content in the product is further decreased, since the bran contains the majority of the grain magnesium. This creates an interesting situation—a trade-off between decreasing phytate and retaining sufficient magnesium in food products. Magnesium bioavailability tends to be low in grains. But certain processing techniques, like fermentation, may help with Mg absorption [113]. Fermentation helps to break down the phytate up to ~ 85% as demonstrated by Nsabimana et al. [119], thereby reducing its inhibitory effect and enhancing the nutritional value of grain-based foods.

## 6. Biofortification Experiments

Many studies have shown that applying Mg fertilizer to Mg-deficient soil improves crop yield and quality. Global meta-analysis conducted by Wang et al. [5] demonstrated that magnesium fertilization can increase crop yield and quality over a wide range of environmental conditions. There are several positive effects of fertilization which are mainly associated with photosynthesis efficiency, enhanced assimilate transport and better nutrient balance, especially with soils with low Mg content.

Biofortification is strategy to enhance concentrations of mineral elements in edible parts of crops through fertilization. It has been recognized as an effective approach to improve crop quality. Fertilization techniques can include nano-fertilizers which have shown a promising development in biofortification due to the significant improvement of nutrient availability and uptake efficiency [6]. Salcido-Martínez et al. [120] showed that use of NanoMg fertilizer significantly increased Mg content in green bean plants while maintaining or improving biomass production and yield. These results indicate that optimized Mg fertilization practices, including the use of nano-fertilizers, can enhance both crop productivity and minerals content, highlighting their potential importance for sustainable agricultural production and improved nutritional quality. Comparing biofortification of green bean plants by NanoMg and MgSO_4_, the most effective treatment to enhance growth and magnesium accumulation in green bean plants cv. ‘Strike’ was NanoMg at 200 mg kg^−1^ (see Table 4). It significantly promoted total biomass, yield, and Mg content in fruits by more than 120% in biofortification compared to the control. The consumption of 100 g of NanoMg-biofortified beans would be adequate to meet daily Mg requirements. According to Kumssa [121] magnesium biofortification may also help to mitigate issues like grass tetany in grazing animals.

However, the content of Mg is increased significantly lower in the study conducted by Coelho et al. [122], where the Mg content in biofortified variants of tomatoes (*Lycopersicum esculentum* L.) by foliar-applied MgSO_4_ has risen only by 2.1% in comparison with the control. Another experiment by Abbas et al. [123] with biologically synthesized magnesium oxide (MgO) nanoparticles, using *Osmanthus* flower extract, has demonstrated significant biofortification potential. At a concentration of 500 mg kg^−1^, they notably enhanced seed germination, maize seedling growth, and photosynthetic activity, resulting in more intense plant coloration and higher magnesium accumulation in plant tissues. The nanoparticles were effectively transported to various plant parts, thereby supporting plant nutrition and overall vitality. These findings confirm the applicability of MgO nanoparticles as efficient nanonutrients for crop biofortification.

There were several research studies addressing magnesium deficiencies in rice, conducted in China. Rice is a staple crop in China and many developing countries. It supplies most of the daily caloric intake. The problem is that the nutritional value of rice, particularly in magnesium, is rather low, often reduced by post-harvest processing and the consumption of polished grains. This contributes to a lack of Mg and other essential nutrients in the population. Since rice plays a crucial role in magnesium intake, enhancing its magnesium content through agricultural practices or biofortification could significantly benefit public health [124,125].

Application of Mg fertilizer has considerably improved rice yield and profitability in Fujian Province. Across 19 field trials, rice yield increased by up to 7.2% and net profit by up to 5.5% with MgO application rates of 15–45 kg ha^−1^. The optimal economic dosage was determined to be MgO 31.6 kg ha^−1^, with adjustments based on soil exchangeable Mg levels. Soils with <80 mg kg^−1^ exchangeable Mg showed the greatest response, confirming widespread Mg deficiency and the importance of tailored Mg fertilization for sustainable rice production [125]. Positive results with Mg fertilizers were also shown in an experiment by He et al. [126], where Mg fertilization significantly improved rice yield, nutrient uptake, and grain quality, even in soils with relatively sufficient native Mg supply. The study demonstrated that Mg acts mainly by enhancing N and P utilization efficiency, rather than simply increasing plant Mg concentration.

There are several crops which are promising for Mg biofortification due to their physiological traits and dietary importance. Leafy greens such as spinach, Swiss chard, and kale naturally have high Mg content and they are common and widely consumed, with potential for Mg accumulation and fast plant growth [97]. Legumes like beans, lentils, and chickpeas also show good Mg content and are protein-rich, with the additional advantages of long storage life and being staple crops in many regions [127]. The most important group, however, are cereals such as wheat, rice, and maize, which are strategic targets for biofortification. Their Mg content is low, especially in the edible endosperm, and further reduced by milling. Since cereals are global dietary staples, they represent key targets for Mg biofortification [128].

## 7. Breeding and Genetic Modification

Another approach to fortification is breeding and genetic modification. That includes developing or breeding plant varieties more effectively in capturing, transmitting, and storing magnesium. It is a technique that utilizes natural or induced gene mutation or natural genetic diversity to enhance magnesium control in roots, leaves, and seeds [88,116]. Biofortification, especially through genetic engineering, presents a strategy to enhance the nutritional value of staple crops by increasing the levels of nutrients, vitamins, and minerals [129]. Recent studies highlight the potential of engineering Mg transporters such as MRS2/MGT to improve magnesium allocation to seeds and leaves [21]. This method ensures that the greatest genetic potential of the plant can be used to maximize nutrient uptake into the plant [130].

Transporters: Strong scientific evidence points out that magnesium transporters play a crucial role in magnesium uptake for various plants, including staple crops like maize and rice. The MGT/MRS2 family, with protein transporters like MGT2, MGT3, and OsMGT1 are involved in uptake and translocation of Mg within the plant. When these genes are forced to overexpress, it can significantly increase Mg uptake and plant tolerance to Mg-deficient conditions [73,131].

Natural variation and QTL: Studies using genome-wide association (GWAS) and quantitative trait loci (QTL) mapping have shown quite significant genetic variety in efficiency of Mg uptake and Mg stress toleration. By comparing many accessions, researchers can pinpoint genomic regions which show desirable traits. This can be furthermore used by breeders to select varieties with stronger Mg uptake and stress tolerance [132,133].

Gene editing: CRISPR/Cas9 technology can be used to increase the content of minerals in crops [134]. This technology has been used to edit the magnesium chelatase gene in sugarcane, a crop with a complex, polyploid genome. This experiment affected leaf color and proved that CRISPR can efficiently target important magnesium-related genes even in plants which are hard to edit [135].

## 8. Serpentin—Possible Source of Magnesium?

Serpentinite could be a new, alternative, and promising source of Mg, with various studies highlighting its potential applications in agriculture and mineral extraction. Global serpentinite reserves are estimated to be in the hundreds of millions of tons, with large deposits located in the USA, Canada, Brazil, Australia, Russia, Armenia, New Zealand, and Paraguay. In Brazil alone, extractable serpentinite reserves amount to 158 million tons [136]. In Europe, serpentinite-rich locations can be found in regions of Italy (the Alps and the Apennines), Austria, Switzerland, France, and Spain [137,138].

Serpentine soils, which form through the weathering of ultramafic rocks—primarily ophiolitic serpentinites—commonly contain chromium (Cr), whose concentration often exceeds 200 mg kg^−1^ significantly higher than those found in non-serpentine soils. This is due to the high chromium content in the minerals, although the content varies greatly between different mineral sources. For example, the chromium content in serpentinite from New Caledonia ranges from 827 to 9528 mg kg^−1^, with nickel (Ni) over 1000 mg kg^−1^ [139]. In India, chromium content ranges from 303 to 4437 mg kg^−1^, and nickel ranges from 1740 to 8033 mg kg^−1^ [140].

Chromium in serpentine is mostly present in the form of Cr_2_O_3_ bound within structural minerals such as spinels and pyroxenes. Its solubility is poor, almost nonexistent; therefore, there is a very low risk of high mobility and toxicity in the soil environment [141]. On the other hand, nickel is more mobile, and up to 10% of total nickel may be phytoavailable, especially under the influence of manganese oxides and amorphous iron oxides, so it poses a certain risk [142].

If we want to use serpentine as a fertilizer, we have to consider its origin and conduct chemical analyses for possible toxic effects of high concentrations of Ni or Cr. Based on the results of these analyses, we can carefully adjust the dose of fertilizer, as some plants are known to hyperaccumulate these metals, which can pose a certain risk. However, other crops, such as grapevines grown in Serbia, have demonstrated a different pattern. Their roots accumulate large amounts of Ni and Cr, but the concentrations in the fruit remain low, minimizing the potential food safety risk [143].

Serpentinite typically contains between 18 and 35% magnesium oxide (MgO), although this can vary based on its source and how the material is processed. This magnesium-rich rock not only serves as a soil remineralizer but also facilitates the extraction of magnesium through mineral carbonation processes. The efficiency of magnesium extraction is influenced by the mineral composition of serpentinite; varieties with higher concentrations of chrysotile and lizardite result in greater magnesium recovery compared to those where antigorite is predominant [144,145].

Different research indicates that serpentinite can be utilized for magnesium extraction via mineral carbonation, achieving an extraction efficiency of approximately 80% under optimal conditions using ammonium sulfate as a solvent.

Extraction of Mg from serpentinite can be done by several methods. The most effective appears to be acid leaching. Using HCl or HNO_3_ at elevated temperatures can boost extraction efficiency up to 97% from chrysotile and lizardite under optimal conditions [146]. Another approach is thermochemical processing, where serpentinite is calcinated and fused with ammonium sulfate ((NH_4_)_2_SO_4_). Subsequent treatment with water and ammonia results in high-purity magnesium oxide, with an MgO content of up to 97.5% [147]. If additives like fluorite powder are used, the whole process can be further improved, as it helps with leaching Mg from serpentinite and can potentially reduce costs by facilitating mineral decomposition [148]. A study by Lu and Neelameggham [149] indicates that serpentinite could be utilized as a magnesium source through mineral carbonation followed by extraction using ammonium sulfate as a solvent. Results depend on conditions and serpentinite type, but extraction efficiency can reach up to 80%.

Ground serpentinite has proven to be as effective as conventional magnesium fertilizers, such as dolomitic limestone or Epsom salt, for crops like oil palm, corn, and beans. In a pot experiment conducted by Viana et al. [150], no significant differences in plant dry matter production were observed between treatments using serpentinite and those using dolomitic limestone. Serpentinite successfully provided sufficient magnesium to fulfill the nutritional requirements necessary for healthy growth and development of both corn and bean plants. Błońska et al. [151] observed a significant improvement in root magnesium uptake even in soils with high Mg content and low Ca content following the application of serpentinite.

It mostly meets safety standards for use as a soil remineralizer and does not harm plant growth or yield. Magnesium is released slowly, so higher doses or different application methods may be needed for long-term nutrition [150,152].

## 9. Summary and Conclusions

Magnesium represents a critical yet frequently underestimated element linking soil fertility, crop productivity and nutritional quality, and human health. Increasing evidence indicates that magnesium deficiency is emerging simultaneously in the plant–soil–human continuum, which highlights magnesium as a key limiting factor.

Agronomic biofortification is an effective and readily applicable strategy to enhance magnesium concentration in crops. Conventional soil Mg fertilization as well as foliar application remains essential, while emerging approaches like nano-fertilizers show potential to improve nutrient-use efficiency. However, their long-term environmental and health impacts require further evaluation. Even alternative sources such as serpentinite may support sustainable magnesium management as slow Mg-releasing fertilizers.

Magnesium biofortification cannot be considered independently, but it is necessary to consider balanced fertilization, soil pH, tillage, Mg bioavailability (especially to newly bred crop varieties), and Mg contents in different parts of plants. Optimizing Mg management therefore represents an important step toward sustainable agriculture and improved global nutritional security.

## Figures and Tables

**Table 1:** Average Mg content in various plant species.

Species	Part	Content in DM (g kg^−1^)	Fertilizing	Source
*Zea mays*	Leaf	~1.4–2.2	N, P, K	[26]
	Grain	~1.6–2.5	-	[27]
*Oryza sativa*	Leaf	~1.22	-	[28]
	Grain	~1.61	-	[29]
*Triticum aestivum*	Leaf	~2.7		[30]
	Grain	~1.1–2.3		[31]
*Phaseolus vulgaris*	Leaf	~4–8	P, K, Mg	[32]
	Grain	~1.6–3.3	-	[29]
*Lupinus* ssp.	Leaf	~1.85–2.92		[33]
	Seed	~1.2–2.5		[34]
*Galega orientalis*	Upper biomass	~2.2–2.8	N, P, K, Ca	[35]
*Trifolium pratense*	Upper biomass	~3.30	-	[36]
*Eruca sativa*	Leaf	~2.8–3.4	Ascorbic acid	[37]
*Lactuca sativa*	Leaf	~3.0–5.7	N, Mg	[38]
*Spinacia oleracea*	Leaf	~3.1–5.1	MgSO_4_	[39]
*Vitis vinifera*	Leaf	~2.0–2.5	N, P, K, manure	[40]
	Fruit (no seed)	~0.2–1.6	-	[41]
*Lycopersicum esculentum*	Leaf	~3.1–3.9	MgSO_4_	[42]
	Fruit	~4.05–4.78	MgSO_4_	
*Camelia sinensis*	Leaf	~2.0–4.3	Mg	[43]
*Mentha* sp.	Leaf	~1.1–1.62	-	[44]

**Table 2:** Representative bioavailable soil Mg contents across continents (average values from different studies).

Continent	Location	Dominant Soil (WRB)	Content (mg kg^−1^)	Dominant Bioavailability	Extraction Method	Source
Africa	Zimbabwe	Lixisols	~27	Low	NH_4_OAc	[79]
America	USA-Iowa	Mollisols	~200–600	High	NH_4_OAc	[78]
	USA-Alabama	Ultisols	~20–120	Medium	NH_4_OAc	[78]
	Brazil	Oxisols	~25–121	Low	NH_4_OAc/KCl	[81]
Asia	China (North)	Chernozems	~275–331	High	NH_4_OAc	[74]
	China	Acrisols	~65–133	Low	NH_4_OAc	[74]
Australia	North-east	Ultisols, Alfisols	~43	Low	NH_4_OAc	[83]
Europe	France (Alsace)	Luvisols	~145–150	High	CAL	[84]
	France (Bretagne)	Cambisols	~60–80	Low-Medium	CAL	[84]
	Spain	Leptosols	~40–500	Medium	NH_4_OAc	[77]
	Czech Republic	Cambisols	~169	Medium	Mehlich 3	[82]

**Table 3:** Overview of commonly used mineral fertilizers containing Mg (modified after Ludemann et al. [101]).

Type	Chemical Formula	Use
Dolomite	CaMg(CO_3_)_2_	For acidic soils, pH control, Mg^2+^ + Ca^2+^
Kieserite	MgSO_4_·H_2_O	Water soluble, fast release Mg^2+^ + SO_4_^2−^
Magnesium oxide	MgO	Slow release, for acidic soils, pH contr.
Magnesium Silicates	various	Very slow release
NPK + Mg	various	Basic fertilization
Ca-Ammonium Nitrate	NH_4_NO_3_ + CaMg(CO_3_)_2_	Basic fertilization, neutral acidity

**Table 4:** Biofortification degree of different fertilizing systems for green bean (in %) [120].

Treatment and Dose (mg kg^−1^)	Biofortification Degree
Control	-
MgSO_4_ 50	70.8 ^d^
MgSO_4_ 100	87.5 ^bc^
MgSO_4_ 200	91.7 ^b^
NanoMg 50	79.1 ^cd^
NanoMg 100	83.3 ^bc^
NanoMg 200	120.8 ^a^

The different letters are meaning significant difference among treatments at *p* < 0.05 (LSD test).

## Data Availability

No new data were created or analyzed in this study.
